# Supplementary figures and images for: Bayesian workflow for time-varying transmission in stratified compartmental infectious disease transmission models
Source: PLoS Comput Biol. 2024 Apr 29;20(4):e1011575. doi: 10.1371/journal.pcbi.1011575 (PMC11081492; doi:10.1371/journal.pcbi.1011575)

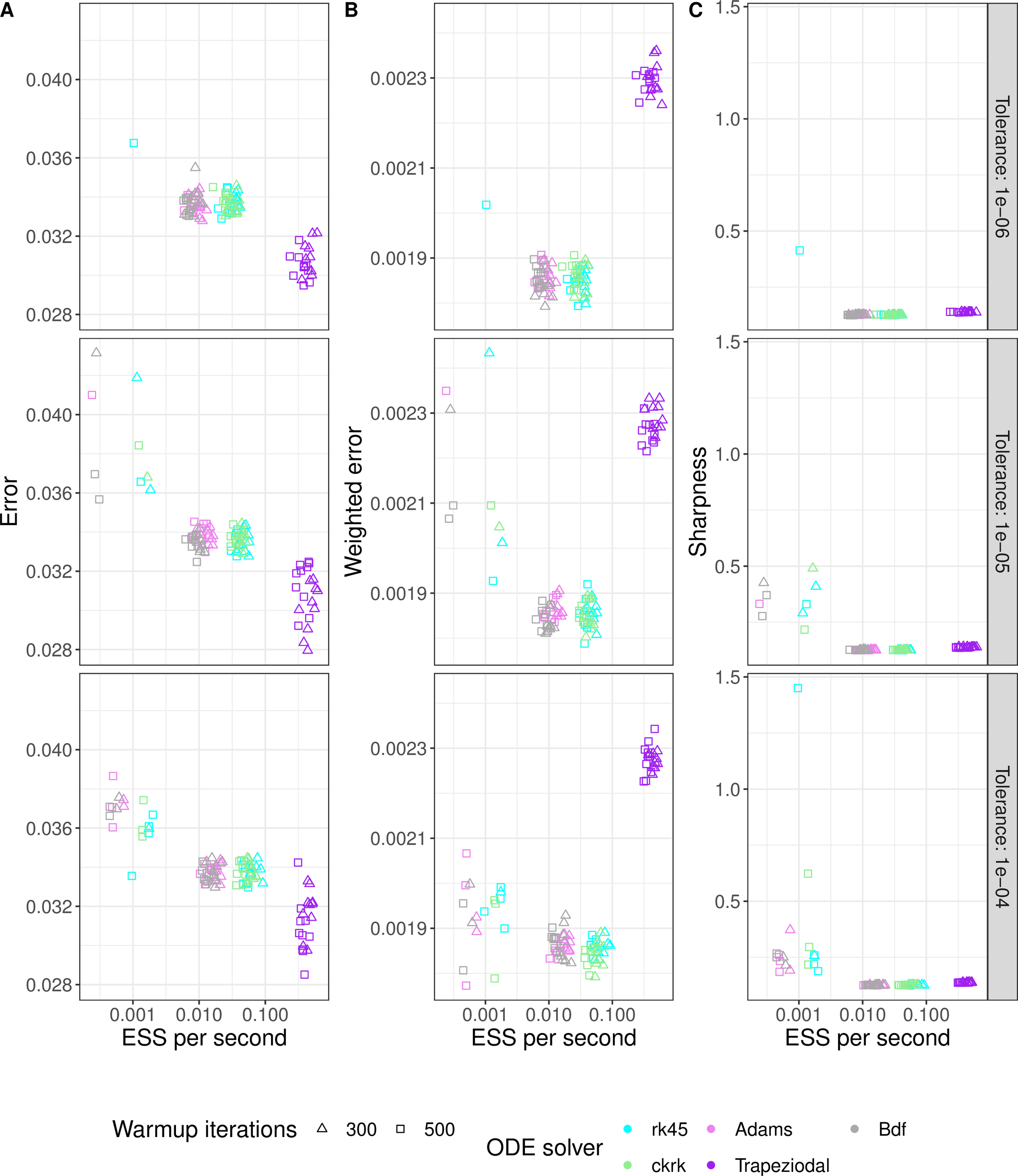

Supplement: S1 Fig — Comparison of computational performance for the Brownian motion model of the time-varying transmission rate of SARS-CoV-2 for simulated, non-stratified data for various tuning parameters: tolerance, ODE solver and number of warm-up iterations. (A) The root mean square error (RMSE) in estimating the time-variation in the transmission. (B) The RMSE weighted by the number of laboratory-confirmed cases per week. (C) The sharpness (size of the 90% confidence interval) of the time-variation in the transmission. (TIF) [file pcbi.1011575.s001.tif]

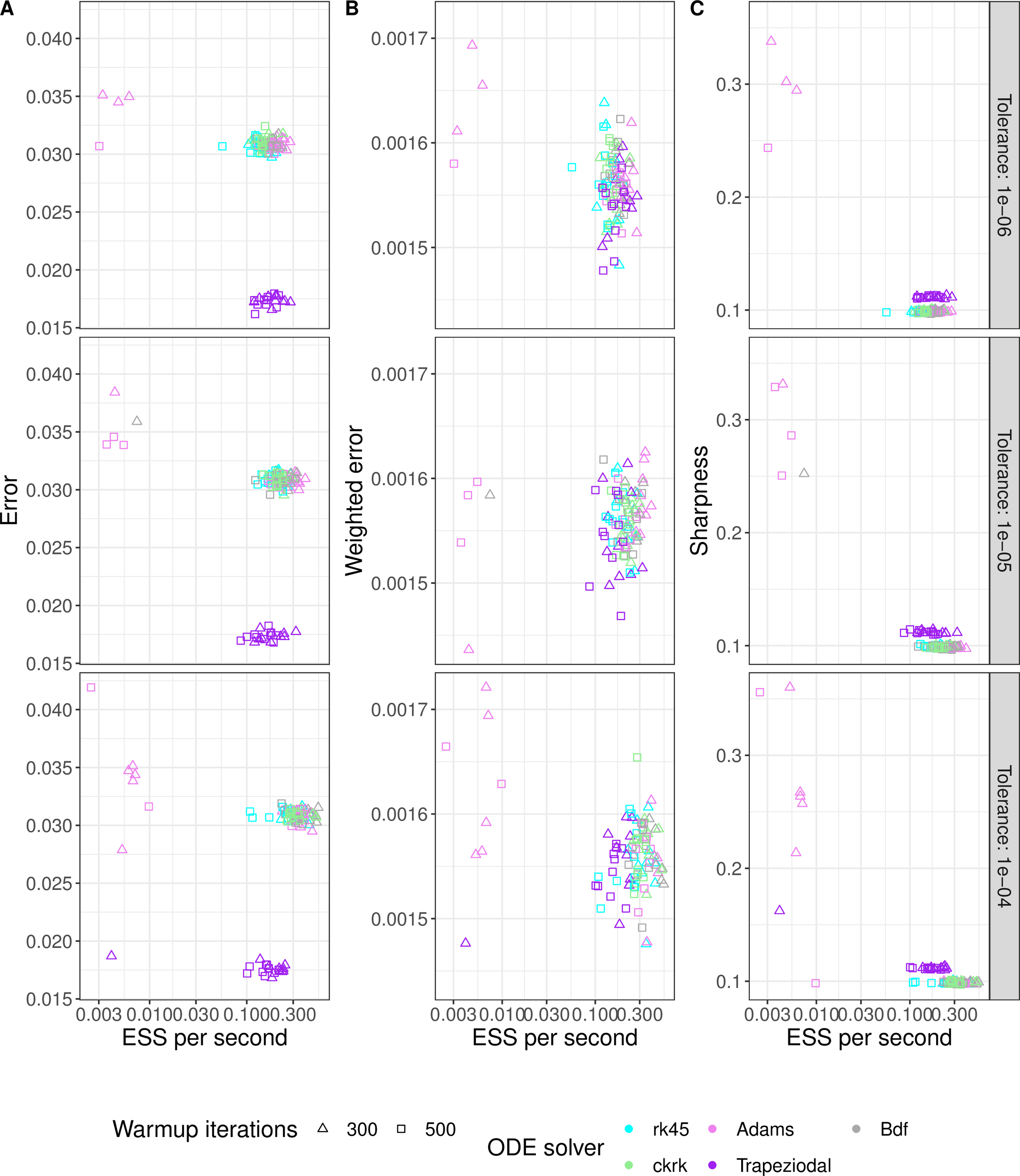

Supplement: S2 Fig — Comparison of computational performance for the B-spline model of the time-varying transmission rate of SARS-CoV-2 for simulated, non-stratified data for various tuning parameters: tolerance, ODE solver and number of warm-up iterations. (A) The root mean squared error (RMSE) in estimating the time-variation in the transmission. (B) The RMSE weighted by the number of laboratory-confirmed cases per week. (C) The sharpness (size of the 90% confidence interval) of the time-variation in the transmission. (TIF) [file pcbi.1011575.s002.tif]

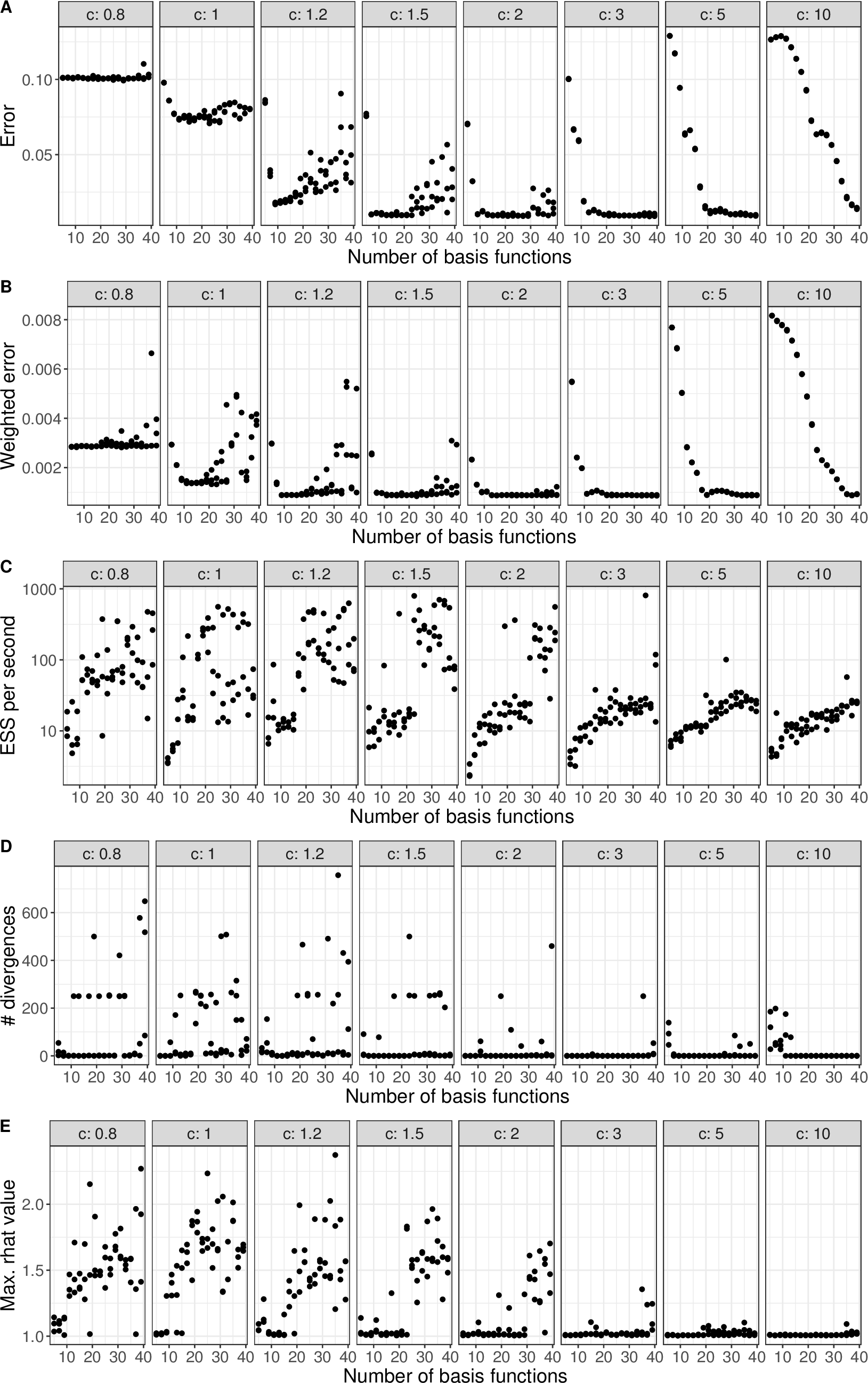

Supplement: S3 Fig — Analysis of the optimal number of basis functions and boundary factor for the approximate Gaussian Processes based time-varying transmission model of SARS-CoV-2 using simulated data. The number of warm-up and sampling iterations are both fixed to 300 and the trapezoidal solver is used. (TIF) [file pcbi.1011575.s003.tif]

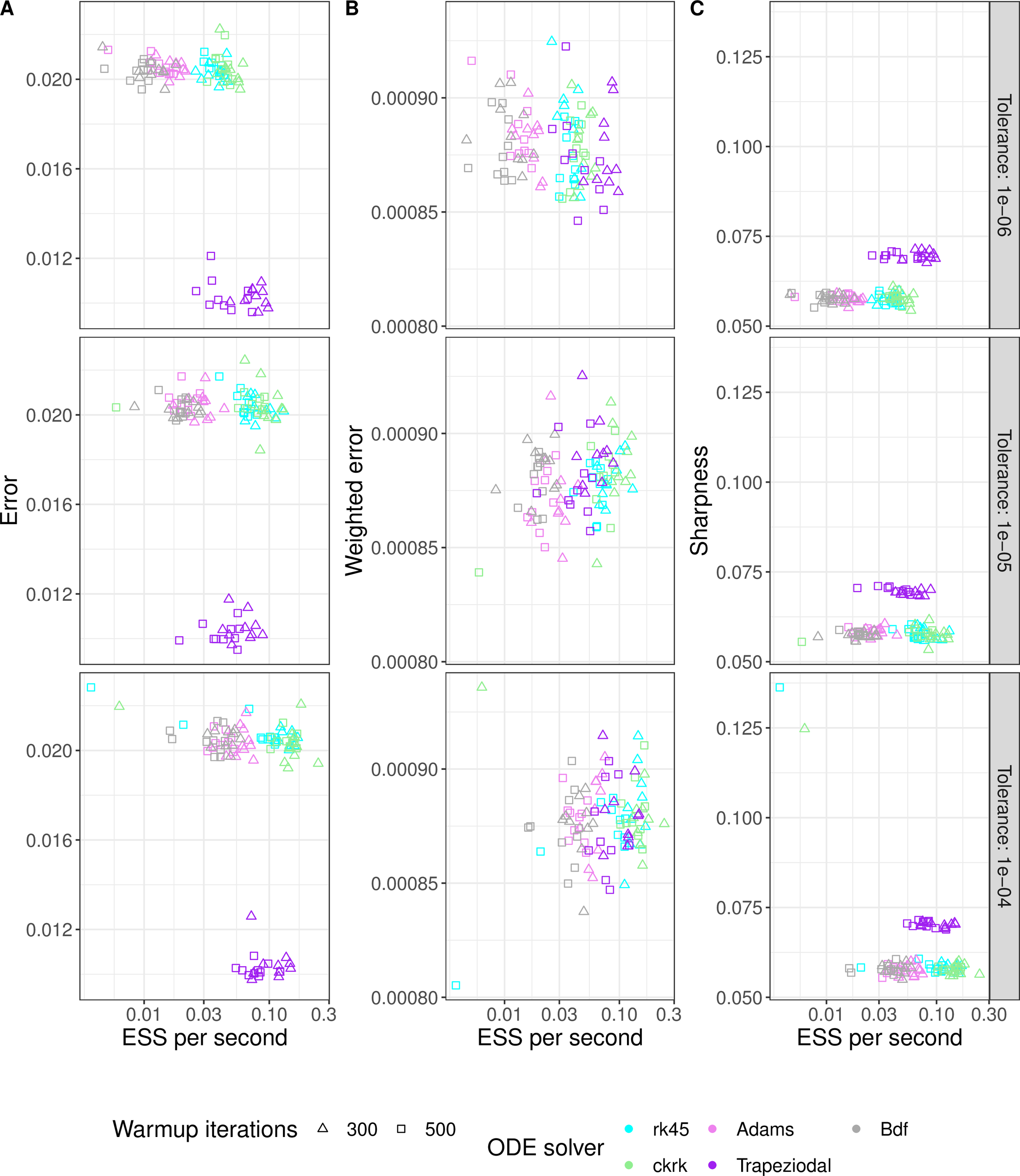

Supplement: S4 Fig — Comparison of computational performance for the approximate Gaussian processes model of the time-varying transmission rate of SARS-CoV-2 for simulated, non-stratified data for various tuning parameters: tolerance, ODE solver and number of warm-up iterations. (A) The root mean squared error (RMSE) in estimating the time-variation in the transmission. (B) The sharpness (size of the 90% confidence interval) of the time-variation in the transmission. (TIF) [file pcbi.1011575.s004.tif]

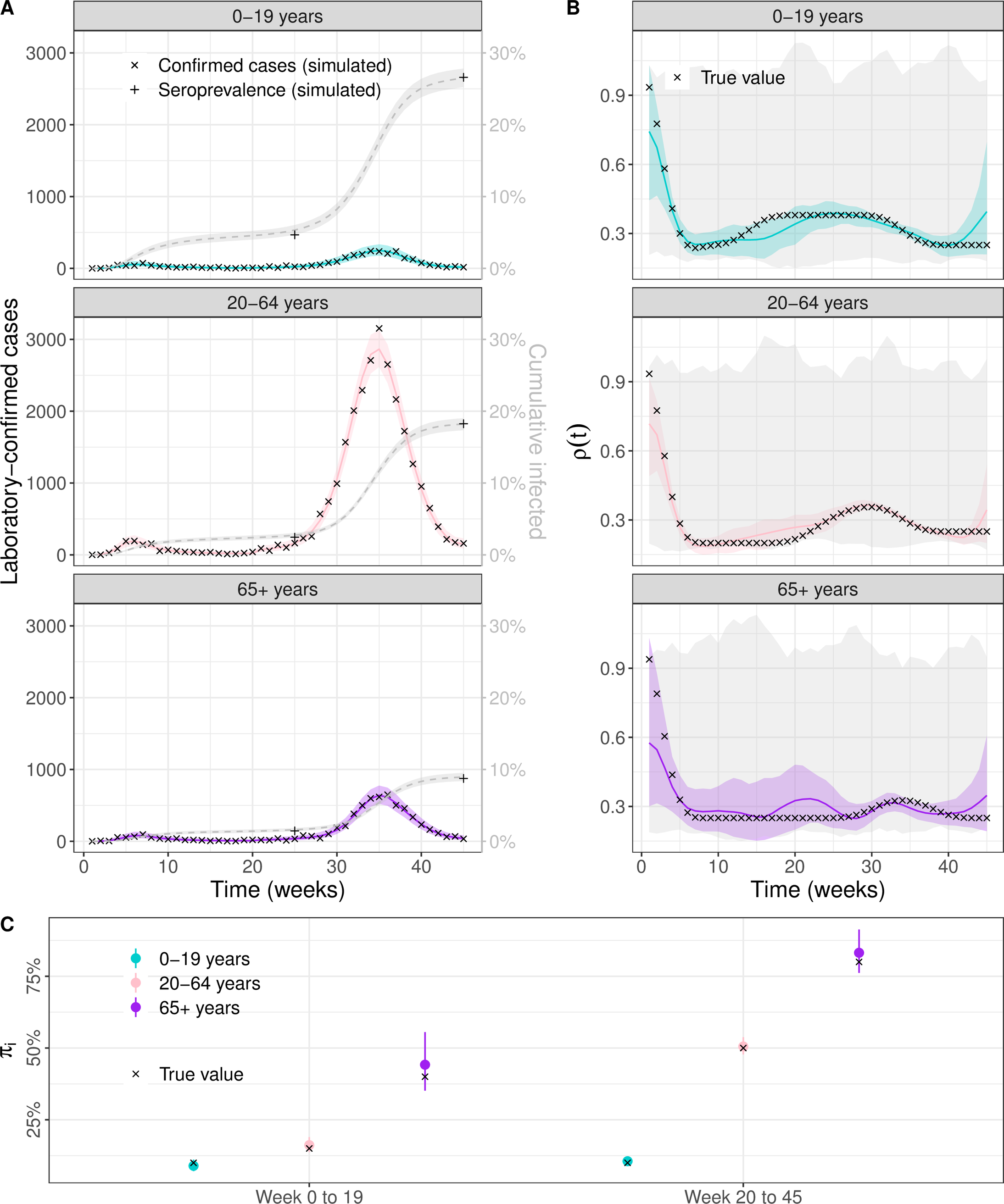

Supplement: S5 Fig — (A) Posterior predictive plot for laboratory-confirmed cases (left y-axis, colored ribbon) and cumulative incidence (right y-axis, gray ribbon) per age group using the B-spline based age-stratified model applied to simulated data. Crosses are weekly simulated counts of laboratory-confirmed cases and pluses are simulated estimates of seroprevalence at two time points. (B) Estimates of the time-varying change in transmission rate per age group using B-splines. Crosses represent the true, simulated values. (C) Estimates of the ascertainment rate per age group and time period. Crosses represent the true, simulated values. (TIF) [file pcbi.1011575.s005.tif]

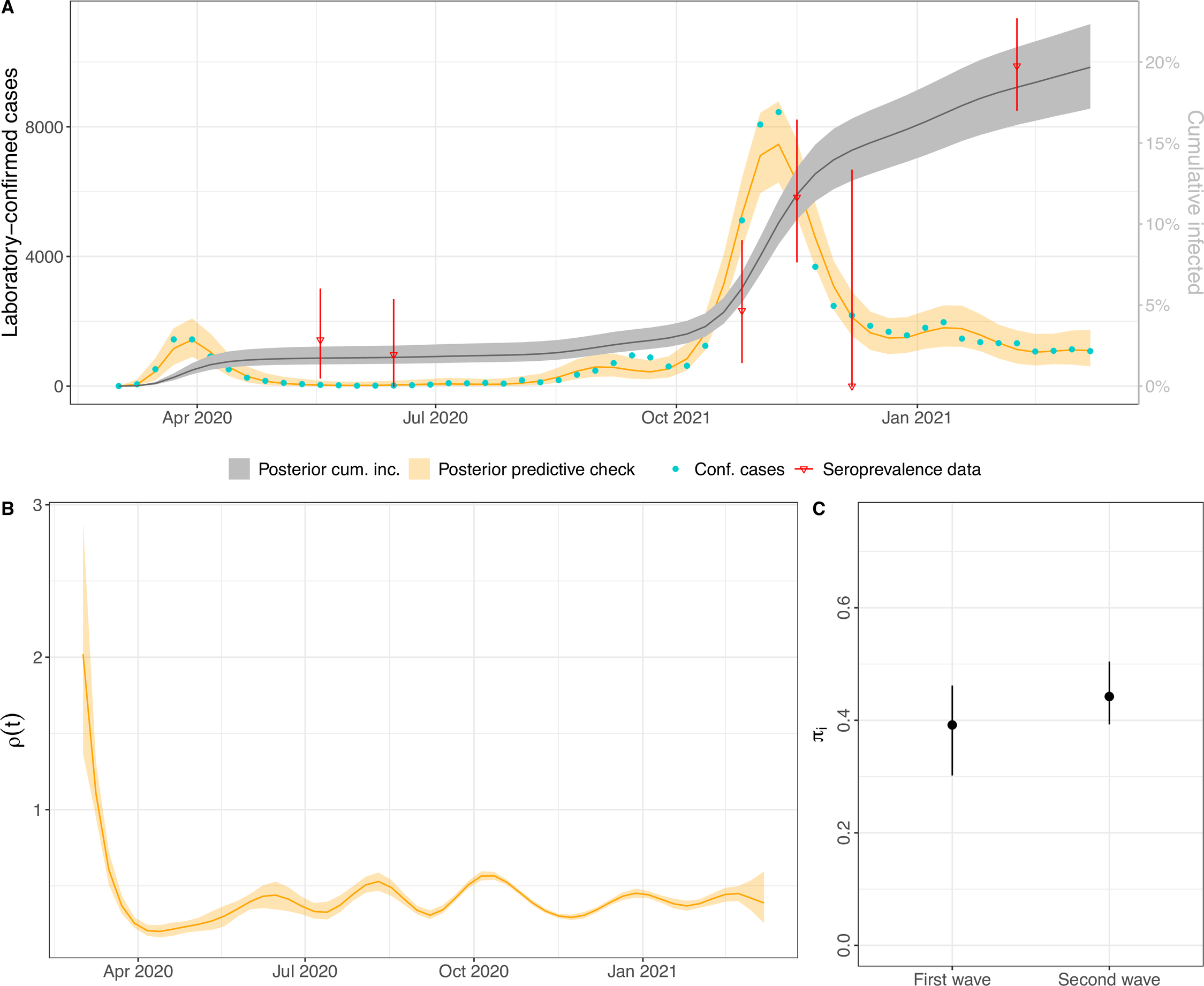

Supplement: S6 Fig — (A) Posterior predictive plot for laboratory-confirmed cases (left y-axis, orange ribbon) and cumulative incidence (right y-axis, gray ribbon). Green circles are weekly counts of laboratory-confirmed cases and red triangles show monthly seroprevalence estimates from data. (B) Estimates of the time-varying change in transmission rate using B-splines. (C) Estimated ascertainment rates for first and second wave. (TIF) [file pcbi.1011575.s006.tif]

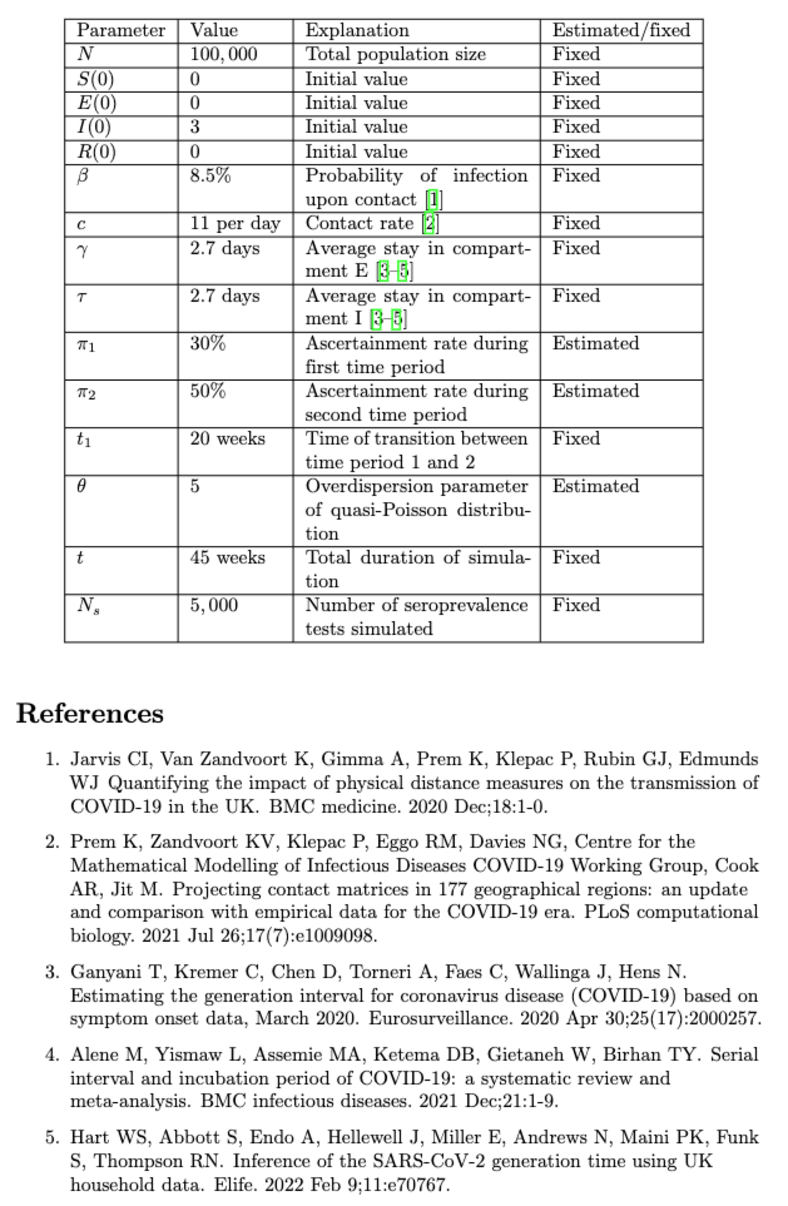

Supplement: S1 Table — (TIF) [file pcbi.1011575.s007.tif]

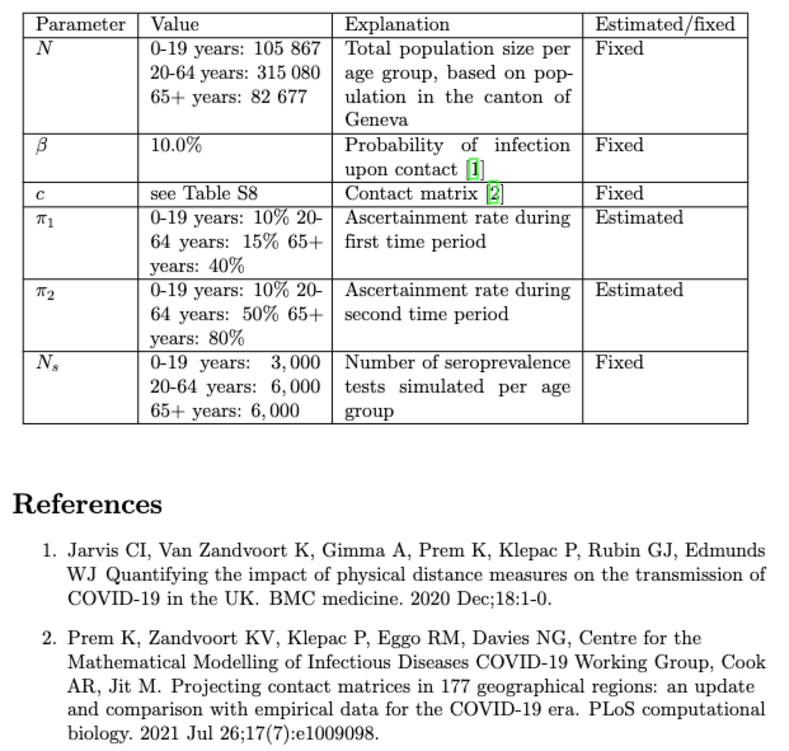

Supplement: S2 Table — (TIF) [file pcbi.1011575.s008.tif]

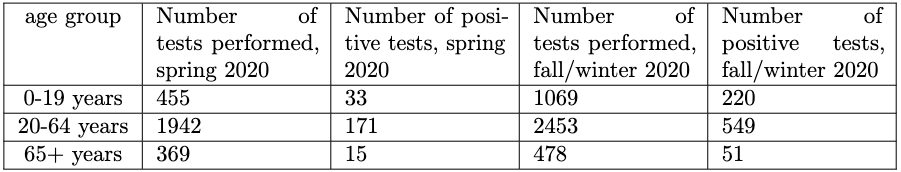

Supplement: S3 Table — (TIF) [file pcbi.1011575.s009.tif]

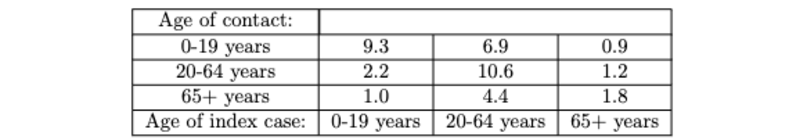

Supplement: S4 Table — This matrix is constructed using Prem et al (2021) and adjusted for the population structure of the canton of Geneva [42]. This matrix is used both for simulating stratified SARS-CoV-2 data and for analysing the data from the canton of Geneva. (TIF) [file pcbi.1011575.s010.tif]
