# Supplementary material for: Bayesian workflow for time-varying transmission in stratified compartmental infectious disease transmission models
Source: PLoS Comput Biol. 2024 Apr 29;20(4):e1011575. doi: 10.1371/journal.pcbi.1011575 (PMC11081492; doi:10.1371/journal.pcbi.1011575)
Supplement: S1 Text — (PDF) [file pcbi.1011575.s011.pdf]

S1 Text

Supplementary methods

Age-stratified SEIR transmission model The system of ODE-equations below represents the age-stratified version of the SEIR transmission model. The subscript for S, I, E, R, N and $\rho(t)$ indicates the age group.

$$\frac{dS_i}{dt} = -\beta\rho_i(t)\sum_{j=1}^{3} c_{ij}S_i\frac{I_j}{N_i}$$
$$\frac{dE_i}{dt} = \beta\rho_i(t)\sum_{j=1}^{3} c_{ij}S_i\frac{I_j}{N_i} - \tau E_i$$
$$\frac{dI_i}{dt} = \tau E_i - \gamma I_i$$
$$\frac{dR_i}{dt} = \gamma I_i$$

Definition of priors We chose a weakly informative gamma prior on the initial basic reproduction number (R_0) of the model, where we set the mean and variance to 2.5 (shape =2.5 and scale = 1, corresponding to an expected R_0 between 0.4 and 6.4 based on the 2.5% and 97.5% percentiles). This basic reproduction number then defines the initial probability of transmission by the relationship derived from the ODE system:$\beta_0 = \frac{R_0 * \gamma}{c}$, where c and γ are assumed to be fixed and known. For the initial number of infected individuals ($I(0)$), we also took a gamma distribution (mean = 0.25, variance=0.25). For the ascertainment rate, we took a beta(2,2) distribution. The prior for the overdispersion parameter of the quasi-Poisson model (θ) is exponentially distributed with mean 0.1. For the negative binomial model, we use a exponential distribution with $\lambda = 1$ as the prior on the inverse of the dispersion parameter (ϕ).

Priors specific for Brownian motion model The priors on the parameters specific for the Brownian motion model are defined such that the weekly transmission rate (η_{weekly}) is distributed as $\eta^{i}_{weekly} \sim norm(\eta^{i-1}_{weekly}, \sigma_{BM})$. Where the prior on BM

is a normal distribution with a zero mean and standard deviation equal to 0.1.

Priors specific for B-splines model For the spline model, we took a gamma prior with shape equal to 2.5 and scale equal to 5, resulting in 0.083 and 1.28 as the 2.5% and 97.5% percentiles.

Priors specific for approximate Gaussian processes model The regression weights are standard normally distributed. The prior on the length scale parameter is a normal distribution with mean equal to 0 and a standard deviation of 3 and for the marginal variance we use an exponential distribution with a rate parameter of 5.
