# Supplementary material for: Bayesian workflow for time-varying transmission in stratified compartmental infectious disease transmission models
Source: PLoS Comput Biol. 2024 Apr 29;20(4):e1011575. doi: 10.1371/journal.pcbi.1011575 (PMC11081492; doi:10.1371/journal.pcbi.1011575)
Supplement: Attachment — Submitted filename: PCB_Bouman2023.pdf [file pcbi.1011575.s012.pdf]

The authors present a Bayesian workflow (also produced as an R package called HETTMO) for fitting a compartmental model of infectious disease transmission that combines four distinct features: 1) accounting for incomplete case ascertainment, 2) a sampling distribution for confirmed cases, 3) time-varying transmission, and 4) age stratification. Different implementations of the workflow are calibrated to SARS-CoV-2 data from Geneva, Switzerland and compared to illustrate how to decide how to implement features 2 and 3 (in terms of relative accuracy and speed). Overall, the manuscript is clear, easy to read, and the results are presented in a way that are easy to understand. However, I had a couple lingering questions.

Lines 18-20 aim to justify and explain the benefits of using a workflow, but I was not sufficiently convinced by their claims. The benchmarking that was done by the authors seems to be very specific to the data that the framework was calibrated to, and it is easy to imagine a case in which the user of the framework would have to select a different implementation. In that case, why not just write out their own model instead of using an R package? There needs to be a better explanation and justification for the need for this type of workflow. Additionally, who is the audience for the workflow? Most infectious disease modelers are equipped with the skills to write and calibrate a compartmental model like the one presented without the need for an R package. Therefore, if not infectious disease modelers, who are the intended users of the package? It’s unclear to me that this R package, or workflow, will ever be used. Maybe some evidence of the rising growth or usage of such workflows could be presented here.

In addition, as alluded to above, this workflow is only calibrated to one very specific dataset. This limitation is mentioned briefly in the discussion. However, I would really like to see at least one other instance of this workflow being used for another infectious disease, to indicate that this workflow is indeed generalizable beyond the very specific circumstances surrounding the COVID-19 pandemic. For example, a past flu season could be a useful set of data for such an exercise.
