# Supplementary material for: Bayesian workflow for time-varying transmission in stratified compartmental infectious disease transmission models
Source: PLoS Comput Biol. 2024 Apr 29;20(4):e1011575. doi: 10.1371/journal.pcbi.1011575 (PMC11081492; doi:10.1371/journal.pcbi.1011575)
Supplement: Attachment — Submitted filename: review_HETTMO.pdf [file pcbi.1011575.s013.pdf]

Reply to reviewers — Bayesian workflow for time-varying transmission in stratified compartmental infectious disease transmission models

We thank the editor and reviewers for evaluating our manuscript. In this document, we reply to the concerns of the reviewers in a point-to-point manner. The replies are colored in red (and italic) and the revised parts of the manuscript are added in blue. The referenced line numbers correspond to the lines in the revised document.

Reviewer #1:

In their manuscript, Bouman and coauthors present a Bayesian workflow to infer time-varying parameters in stratified compartmental models. They identify four key features within this framework that need to account for i) incomplete case ascertain (a.k.a., case underreporting), ii) adequate sampling for lab-confirmed cases, iii) flexible implementation of the time-varying transmission rate, and iv) age structure. They explore different models for each and variations of the parameters thereof and validate the workflow using both synthetic and real-world data (first two COVID-19 waves in the canton of Geneva, CH). They conclude that the best-performing methods (for the disease considered and dataset quality) are quasi-poisson for sampling and BSplines for the time-varying transmission rate.

Besides providing valuable insights from the dataset they analyze (which, although regional, is novel), the article is very educational and open with the methodologies applied -- the kind of paper one would give their students to explore a new method. While I am a big fan of such papers, we have some reservations (which we list below) on the generality of the approach proposed.

We thank the reviewer for their summary of our manuscript and we are delighted that the reviewer mentions the main points we aimed to convey with our study.

Major

1. On the generalizability of the proposed approach. While the paper, title and abstract are presented and discussed in a general way, the choice of a SEIR model (and other points listed below) are already assumptions strong enough to constrain its applicability considerably. This goes beyond the choice of parameters, which could help us translate from very similar diseases or variants thereof; it's about mechanisms. Other common respiratory viruses, as e.g. the respiratory Syncytial virus, have markedly different dynamics and thus require markedly different structures (e.g., an SIS model in cascade like that of Zheng and coauthors in JAMA Open 2021). Here, incidence and seroprevalence would have to be re-defined in terms of the new compartments and the timescales involved considered when choosing which functions to use to match the data. Some non-pharmaceutical interventions require extra compartments, and testing could also be thought of as an intervention that reduces transmissibility (as test-trace-quarantine). Vaccination and pharmaceutical interventions might not be relevant when analyzing historic 2020 COVID-19 data, but they should be when analyzing influenza. That being said, the methodology is presented in a way that is general enough to be easily adapted to account for the points I mentioned before -- please expand the methods on how to adapt the workflow to other diseases (i.e., other compartments and transition rules) and rephrase where applicable, acknowledging that the current results for real-world and synthetic

data are COVID-specific. Besides, how flexible and intuitive is the R implementation of HETTMO to compartments and mechanisms? [as we are not fluent R users, we couldn't check this point ourselves]

We agree with the reviewer that the generalizability of the methods did not yet get the attention in the manuscript that it deserves. We note that the four features that we highlight in our manuscript do not depend on the specific choice of the underlying compartmental model. The choice of the SEIR model that we presented for COVID-19 indeed limits the exact use-case. In order to exemplify our methodology and present the importance of these four features, we feel that we need to present a specific example. However, the code in the HETTMO package can be easily adapted to different compartmental models by changing a single function. We added the following paragraph to the method section to explain this (p. 12/13, lines 259-272):

Generality of the proposed approach

The HETTMO package includes the scripts and functions for the different model iterations presented in this study, which form the Bayesian workflow together with the model comparison. This workflow is tailored to study the early spread of SARS-CoV-2, justifying the choice of an SEIR model, the different assumptions on how model outputs are linked to the data, and the specific parametrization. Other situations and pathogens would likely require adaptations, such as additional compartments for vaccinated individuals or waning of immunity. Nevertheless, the presented case-study remains relevant in the wider context of infectious disease modeling. The four crucial features that were identified are broadly applicable for modeling many infectious diseases, and our comparisons regarding the most efficient implementations likely apply to other situations. In addition, our workflow can be directly used as a basis for other models, both technically, by providing easily adaptable code, and conceptually, by showcasing the advantages of the Bayesian workflow in this context.

As suggested, we also adjusted text throughout the manuscript to indicate that the presented results are specific for COVID-19.

In this work, we present a Bayesian workflow for a compartmental transmission model to analyze the transmission of SARS-CoV-2 that includes these four essential features.
(lines 58-60)

We release the scripts and functions for the different model iterations presented in this study in an R package called HETTMO (for HETerogeneous Transmission MOdel). The code can easily be adapted to other situations and pathogens, with the objective of promoting and facilitating access to this type of methods and making the process of model validation and comparison insightful.
(lines 65-69)

We present a Bayesian workflow to find an optimal compartmental transmission model aimed at analyzing the transmission of a respiratory virus, with SARS-CoV-2 as a case-study, in a population over a time period short enough so that immunity waning can be ignored (a few months or years).
(lines 274-277)

2. On the model's structure and equations to match to observables. The SEIR engine used for simulation and inference is a strong assumption; it says that the disease that spreads is very COVID-19-like. This is not only because of the similarity with the typical disease stages of COVID (well, neglecting asymptomatic individuals), but also because of the way that seroprevalence and incidence are modelled. Defining the cumulative incidence through the compartment $R(t)$ disregards the individuals that are still in the infectious compartment but have been already tested. This works here because of the short infectious period of COVID (and the much lower estimate the authors use for $1/\gamma$) and the smoothing induced by real-world seroprevalence studies. Furthermore, defining the modelled incidence as the difference between the recovered from one point to the other disregards the newly reported cases, which again average out with the weekly resolution of the data the authors use. While these are valid modelling choices for a COVID-19-like disease, they can be wrong assumptions for other diseases. I recommend expanding the discussions in this regard so that readers are aware of other modelling choices and possibilities.

We thank the reviewer for pointing out that we did not pay enough attention to the SARS-CoV-2 specific choices in the discussion. As suggested, we extended the limitations paragraph in the discussion to address this issue (lines 460-467).

The model parametrization and the way in which the incidence is coupled to the removed compartment is specific for SARS-CoV-2. However, we emphasize that the four crucial features that are the main focus of the Bayesian workflow are central for a broad range of infectious diseases. Our study provides a starting point for extensions relaxing these assumptions and in the method section we describe how the framework can be adapted to other situations and diseases.

3. On the selection of parameters. Not to be repetitive with the issue of COVID-19-like parameters (the authors do a good job discussing this in the paper), the definition of parameters in Table S6 is wrong or flipped; τ should be the latent period, i.e., from infection to before turning infectious, and be the residence time in E. [side note, as τ is typically thought to as with time units, defining it as a rate can be a bit unexpected for some readers and reviewers]. γ is typically the recovery rate, and its inverse is the days that it takes to recover (8--10)?

We are grateful that the reviewer noticed this inconsistency and have corrected table S6 with the correct parameter description.

If simulating COVID, the parameters we had in mind were τ closer to 4--5 days, and $1/\gamma - \tau \approx 4-8$ days.

This reviewer's comment made us realize that we have not explained our parameter choice with enough detail. We agree with the reviewer that a latent period of 4–5 days is more reasonable and that the average time spent in the I compartment is likely longer than in our parametrization. However, we assume that individuals leave the I compartment at the time where they receive a positive PCR test, as, especially during the time-frame we consider (the early phase of the pandemic), individuals are likely to isolate after receiving a positive test. We therefore believe that it is most relevant to model the generation time (estimated to be 5.2

days during the studied time period) correctly, rather than using individual estimates for the latent period and the recovery rate. We added a more detailed explanation about this to the manuscript on lines 84-92.

Both τ and γ are fixed, such that the generation time is 5.2 days, with this time equally distributed between the exposed and infected compartments [33]–[35]. We have chosen to parameterize γ and τ together based on the generation time rather than with independent estimates for the latent period and the recovery rate. The reason is that we assume that individuals will isolate after a positive PCR test during the study period and therefore move to the R compartment earlier than would be expected based on the recovery rate. Moreover, since our study period was less than a year, we could assume that there is no waning of immunity and that the total population size is constant.

The definitions are inaccurate on line 78, as τ (or its inverse) should be _the latent period_ of the disease.

We have adjusted the description of τ accordingly.

Also note that parameters for the contact matrix might not be available nor represent regional differences, and the mechanisms that cause the quasi-Poisson sampling to be the best might be different in other regions or when analyzing other diseases.

We have accommodated the reviewer's suggestion by adding an additional limitation to the limitations paragraph in the discussion (lines 436-440).

Second, our observation that the quasi-Poisson distribution best describes the noise in the ascertainment of laboratory-confirmed SARS-CoV-2 cases is specific to the data reported in the canton of Geneva. For other diseases and regions, an alternative distribution could be more appropriate.

4. On the error function and applicability of the methodology to emerging diseases. In lines 216 and 217, it states that the model fit was performed by computing the RMSE between real and estimated values for the transmission rate $\rho(t)$. But there can be a catch with this, which is also noted in lines 307 and 308: larger deviations occur when the number of cases is low. If that is the case, then small perturbations to the number of tests will have a drastic effect on the trends of ρ, which will turn erratic. As RMSE is agnostic to the time of the measurements, deviations from these noisy measurements will weigh the same as real errors in times of high case numbers (which should be considered more reliable). For the simulated dataset, where transitions are smooth, and for the data analyzed (where high case numbers and the weekly accumulation of cases do the smoothing part) it is not a problem in general. However, how could this be accounted for in the proposed framework? Perhaps through the introduction of weights or by having a different "goal" variable?

We appreciate the suggestion of the reviewer and included an additional error measure to the results to accommodate this. The measure we added is the weighted root mean squared error, where the weights are determined by the number of cases per time point. We added the definition of this measure to the methods (lines 225-229).

We evaluate predictive performance by computing the root mean squared error (RMSE) between the estimated and true value of ρ(t), a RMSE weighted by the number of laboratory-confirmed cases per week, and evaluate computational performance by comparing the number of effective samples per second.

As we did not want to overcrowd our first result figure, we decided to add the comparison of the hyper-parameters with the weighted RMSE to the supplementary figures (S1-S4). With this alternative error measure, we mostly observe a difference for the trapezoidal solver, which performs worse in terms of the weighted error compared with the RMSE. This was to be expected, as the trapezoidal solver does not implement adaptive step sizes. We refer to these extended supplementary figures in the result section. See lines 301-307.

The choice of the solver can result, in some cases, in a factor of 1,000 difference in performance (comparing the ESS per second for Adams and trapezoidal solvers, S1). In terms of absolute error (based on RMSE), the trapezoidal solver performs best for each time-varying model. However, the relative error (based on weighted RMSE) is either higher (Brownian motion, Figure S1) or similar (B-splines and aGPs, Figures S2 and S4). Moreover, with B-splines and aGPs, the average performance (based on ESS per second) is increased by approximately 25%, with the ckrk solver.

5. On short-term forecasts and real-time surveillance data analysis. Although mentioned, the predictive power of the models is not explored. The authors mention that these models that yield results with increasing variance over time are preferred, but this could be assessed with a leave-future-out analysis of the dataset (not necessary in this case, as it is not the point of the manuscript -- but should be mentioned). However, it is not clear to us how to use this approach in real time to estimate π_t; is it necessary to define periods of constant π? How do we decide how many of these we need for a new dataset? How much time should we let pass before estimating π? Discussing these questions could enlighten readers trying to analyze their datasets in the future.

We thank the reviewers for bringing up the application of real-time surveillance, which is indeed relevant in this context. We added a mention of the leave-future-out analysis that could be used to assess the predictive power of the various methods in the discussion (lines 452-453).

We did not compare the precision for short-term forecasting, which could be done with a leave-future-out analysis.

Moreover, we added an explanation of when the methodology could be used for near-real-time surveillance to the discussion as well (429-432).

The applications presented in this study rely on historical (SARS-CoV-2) data. If we can assume that the ascertainment of cases has been constant since the last available seroprevalence study, the methodology could also be used for near-real-time surveillance.

Minor

1. Figure and equations do not include stratification

This was done on purpose to simplify the notation. We added a stratified version of the equations to the supplementary material.

2. Perhaps a demographic-inspired partition of the 18--24 would represent a general case better?

We thank the reviewers for this suggestion and we adjusted our methods to indeed distribute the antibody tests between the age-groups using the age-distribution in the population of Geneva, rather than assuming a uniform distribution. As a result of this change of assumption we have in total 1069 (0-19 years old) and 2453 (20-59 years old) antibody tests in the winter wave of the seroprevalence study instead of 1083 and 2439 respectively. For the number of positive tests we now have 220 (0-19 years old) and 549 (20-59 years old) instead of 224 and 545 respectively. The updated data are shown in Supplementy Table 8. We have reran the analyses accordingly and updated the relevant figures. We also adjusted the text on lines 246-249 in the method section to reflect this change in methodology.

As the second serosurvey uses a different grouping, we reallocated the results in age group 18-24 to age groups 0-19 and 20-59 using the age-distribution in the population of the canton of Geneva as recorded by the Federal Office of Statistics [51]. These data are available in the HETTMO R-package.

3. The author's summary could be less technical and streamlined towards the methodological/educational approach of the paper.

We agree with the reviewer that the author`s summary was too technical. We have rewritten the summary to better represent the main focus of the paper.

Mathematical models are a central tool for understanding the spread of infectious diseases. These models can frequently be fitted to surveillance data such as the number of laboratory-confirmed cases and seroprevalence over time. We identified that in these situations, four crucial features are required for a model to provide insightful information for managing an epidemic. These features relate to the adjustment for incomplete case ascertainment, to the choice of sampling distribution, to the variation of transmission over time and to the stratification by age. For each feature, we identify and compare several implementation options on simulated data. This structural comparison of methods results in a Bayesian workflow that is optimized for modeling the transmission of SARS-CoV-2 over a short period. We highlight the advantages and limitations of our approach in a real situation, using real-world SARS-CoV-2 data from the canton of Geneva. In addition to providing validated solutions to important technical points, such a comprehensive workflow helps to improve the reliability and the transparency of epidemic models.

4. (line 10) COVID-19 pandemic instead of SARS-CoV-2 pandemic? as SARS-CoV-2 was the pathogen causing the pandemic disease.

Thank you for the suggestion, we have adjusted the terminology.

5. About the HET in HETTMO; We are a bit skeptical about how much heterogeneity is captured here, as the core approach remains mean-field.

The reviewer is indeed correct in their observation that the core approach of the presented Bayesian workflow is mean-field. However, the “HET” in HETTMO rather represents the heterogeneity in the population that can be captured with our methodology using stratification. In our example for SARS-CoV-2 we model three age-groups in the population that can capture some of the heterogeneity that is relevant for the spread of SARS-CoV-2. We have added an explanation of the “HET” part of the acronym in the method section (lines 252-254).

HETTMO is the acronym for “heterogeneous transmission model” since the package can be used to model a heterogenous population that is stratified by, for example, age.

6. It is probably a good idea to make the color of the y-axis similar to the line color when using double y-axis (e.g.- Figure 2, the y-axis on the left side will make easier for understand that this axis is for the green line and Grey on the right y-axis for Grey line in the figure)

This is a good suggestion, we indeed implemented the change in y-axis color in figures 2A, 3A and supplementary figure 5.

Reviewer #2:

The authors present a Bayesian workflow (also produced as an R package called HETTMO) for fitting a compartmental model of infectious disease transmission that combines four distinct features: 1) accounting for incomplete case ascertainment, 2) a sampling distribution for confirmed cases, 3) time-varying transmission, and 4) age stratification. Different implementations of the workflow are calibrated to SARS-CoV-2 data from Geneva, Switzerland and compared to illustrate how to decide how to implement features 2 and 3 (in terms of relative accuracy and speed). Overall, the manuscript is clear, easy to read, and the results are presented in a way that is easy to understand. However, I had a couple lingering questions.

We thank the reviewer for their summary and overall positive judgement of our manuscript.

Lines 18-20 aim to justify and explain the benefits of using a workflow, but I was not sufficiently convinced by their claims. The benchmarking that was done by the authors seems to be very specific to the data that the framework was calibrated to, and it is easy to imagine a case in which the user of the framework would have to select a different implementation. In that case, why not just write out their own model instead of using an R package? There needs to be a better explanation and justification for the need for this type of workflow. Additionally, who is the audience for the workflow? Most infectious disease modelers are equipped with the skills to write and calibrate a compartmental model like the one presented without the need for an R package. Therefore, if not infectious disease modelers, who are the intended users of the package? It’s unclear to me that this R package, or workflow, will ever be used. Maybe some evidence of the rising growth or usage of such workflows could be presented here.

We thank the reviewer for bringing up this point. As we believe that a Bayesian workflow such as the one we present in the manuscript can be highly beneficial for the field of infectious disease modeling, we are happy to take the opportunity to explain its benefits here and in our manuscript in more detail. We agree with the reviewer that infectious disease modelers, such as ourselves, are able to write their own model in R. However, the effort that goes into programming and comparing several implementations of various complex features to determine the optimal model is not to be underestimated. The workflow also includes exactly this additional burden of work before an optimal model can be constructed. In our study, we also model time-varying transmission with techniques (B-splines and aGPs as forcing functions) that, to our knowledge, have not previously been implemented within an ODE-based model. Developing and testing these methods is time-consuming, even for experienced infectious disease modelers. Therefore, we believe that this study will be of direct, technical interest to infectious disease modelers. Moreover, the general approach of the Bayesian workflow, with this systematic and iterative model building, has the potential to bring important benefits to the field of infectious disease modelling in terms of reliability and transparency.

We agree with the reviewer that the benefits of the Bayesian workflow could be conveyed better in the manuscript and that the exact meaning of the Bayesian workflow should be presented better. We have made various changes throughout the manuscript to improve this.

In the introduction:

Lines 21-23:
Therefore, there is a growing demand for readily available Bayesian workflows as defined by Gelman et al. (2020), which provide access to models that have been through the iterative process of model building, validation, understanding and comparison [12].

Lines 58-69:
In this work, we present a Bayesian workflow for a compartmental transmission model to analyze the transmission of SARS-CoV-2 that includes these four essential features. To this aim, we assess the statistical accuracy and computational efficiency of several of these model choices, including three sampling distributions (Poisson, quasi-Poisson, and negative binomial) and three methods for implementing a time-varying transmission rate (Brownian motion, B-splines, and approximate Gaussian processes). For these assessments, we use both simulated data and real-world data from SARS-CoV-2 in Geneva, Switzerland. We release the scripts and functions for the different model iterations presented in this study in an R package called HETTMO (for "heterogeneous transmission model"). This code can easily be adapted to other situations and pathogens, with the objective of promoting and facilitating access to this type of methods and making the process of model validation and comparison insightful.

In the results:

Lines 274-277:
We present a Bayesian workflow to find an optimal compartmental transmission model aimed at analyzing the transmission of a respiratory virus, currently SARS-CoV-2, in a

population over a time period short enough so that immunity waning can be ignored (a few months or years).

Lines 289-290:
The next step in the Bayesian workflow is to benchmark several implementations of the time-varying transmission in a simulation study (feature 3).

In the discussion:

Lines 267-371:
The final iteration of the model in the workflow includes four main features deemed as essential for this task: adjustment for incomplete and differential case ascertainment across age groups, adequate sampling distribution, time-varying transmission rate and stratification by age.

Lines 374-377:
We also release the various model versions within the workflow as an out-of-the-box R package, where all model variations are available (https://github.com/JudithBouman2412/HETTMO or https://doi.org/10.5281/zenodo.10619448).

Lines 398-400:
The application of the optimal model suggested by our workflow to the situation of the SARS-CoV-2 epidemic in the canton of Geneva, Switzerland, in 2020 highlights the practical advantages of our proposed approach.

Lines 472-476
By combining the structure of ODE-based compartmental transmission models and the power of full Bayesian inference, the presented Bayesian workflow, and its functionality in the HETTMO package provides such a tool for relatively simple situations: a newly-emerging respiratory virus spreading in a population before vaccination and immunity waning can play a role.

In addition, as alluded to above, this workflow is only calibrated to one very specific dataset. This limitation is mentioned briefly in the discussion. However, I would really like to see at least one other instance of this workflow being used for another infectious disease, to indicate that this workflow is indeed generalizable beyond the very specific circumstances surrounding the COVID-19 pandemic. For example, a past flu season could be a useful set of data for such an exercise.

We thank the reviewer for bringing up the generalizability of the presented methods. Indeed, we calibrated the workflow for SARS-CoV-2. The choice of an SEIR model as the backbone for the methodology is the direct result of assumptions we made specific for SARS-CoV-2. However, the four essential model features we highlighted in our manuscript are not influenced by these specific assumptions. As a result, the workflow can be easily adapted to suit other infectious diseases while keeping the four essential features. We agree with the reviewer that this has not received enough attention in the manuscript. We now extended our methods to describe how the workflow can be adapted to other infectious diseases (lines 259-272).

Generality of the proposed approach

The HETTMO package includes the scripts and functions for the different model iterations presented in this study, which form the Bayesian workflow together with the model comparison. This workflow is tailored to study the early spread of SARS-CoV-2, justifying the choice of an SEIR model, the different assumptions on how model outputs are linked to the data, and the specific parametrization. Other situations and pathogens would likely require adaptations, such as additional compartments for vaccinated individuals or waning of immunity. Nevertheless, the presented case-study remains relevant in the wider context of infectious disease modeling. The four crucial features that were identified are broadly applicable for modeling many infectious diseases, and our comparisons regarding the most efficient implementations likely apply to other situations. In addition, our workflow can be directly used as a basis for other models, both technically, by providing easily adaptable code, and conceptually, by showcasing the advantages of the Bayesian workflow in this context.

The reviewer also suggests extending our workflow to influenza within the current manuscript. However, the main aim of our paper is to benchmark two essential features: (1) the sampling distribution of the laboratory confirmed cases and (2) the method used to implement a time-varying transmission rate, and to present a workflow that includes these features and two additional features: (3) a correction for incomplete case ascertainment and (4) stratification of the population is sub-groups. We find that adapting the workflow to influenza is beyond the scope of our current study, as it would require a very different approach to model influenza with seroprevalence data compared to using seroprevalence data for SARS-CoV-2. SARS-CoV-2 emerged in 2019 and therefore the seroprevalence of the infection during the beginning of the pandemic in 2020 could be equated to the cumulative incidence of the infection. For influenza, on the other hand, the relation between the seroprevalence and the seasonal cumulative incidence is much more complicated. This is because the number of different disease histories an individual could have is extremely large, due to the long history of Influenza infections in humans. As a result, to apply our framework to the transmission of influenza, the requirements to the seroprevalence data are more complicated (it should contain different subtypes, be able to differentiate between antibodies due to vaccination and infection) and the ODE backbone should be adjusted to include the complicated and variable disease histories. We currently do not possess the right type of data to perform such an analysis. Therefore, even though we think this would be a very interesting and worthy exercise, we currently are not able to include such an analysis in our manuscript.
