# Supplementary material for: Bayesian workflow for time-varying transmission in stratified compartmental infectious disease transmission models
Source: PLoS Comput Biol. 2024 Apr 29;20(4):e1011575. doi: 10.1371/journal.pcbi.1011575 (PMC11081492; doi:10.1371/journal.pcbi.1011575)
Supplement: Attachment — Submitted filename: revision_march_HETTMO_JR.pdf [file pcbi.1011575.s014.pdf]

We thank the editor and reviewers for re-evaluating our manuscript. In this document, we reply to the remaining concerns of the reviewers in a point-to-point manner. The replies are colored in red (and italic), and the revised parts of the manuscript are added in blue. *The referenced line numbers correspond to the lines in the revised document.*

Reviewer #1: Thank you for the consideration of our suggestions. We are satisfied with the revisions and have no further observations.

We are glad to read that the reviewer is satisfied with our previous revision, and we thank the reviewer for their time.

Reviewer #2: I appreciate the fact that the authors responded to comments and further explained their reasoning. However, I still think the authors should potentially perform a second analysis using their R package on a different set of COVID-19 data to further show the robustness of their analytical approach.

We are grateful that the second reviewer took time to re-evaluate our manuscript. We appreciate the suggestion to apply our R package to a different set of COVID-19 data to further show the robustness of our approach.

We added a second example of our model. In this example we apply the unstratified B-spline based model to laboratory-confirmed cases of the canton of Vaud, Switzerland. Seroprevalence data was collected by the Corona Immunitas Research group and published in Tancredi et al. (2023). We acquired permission for using their data and publishing our results. We hope that with this second analysis can take away any remaining concerns about the approach.

The results of this second example are shown in S6 Fig.

S6 Fig.

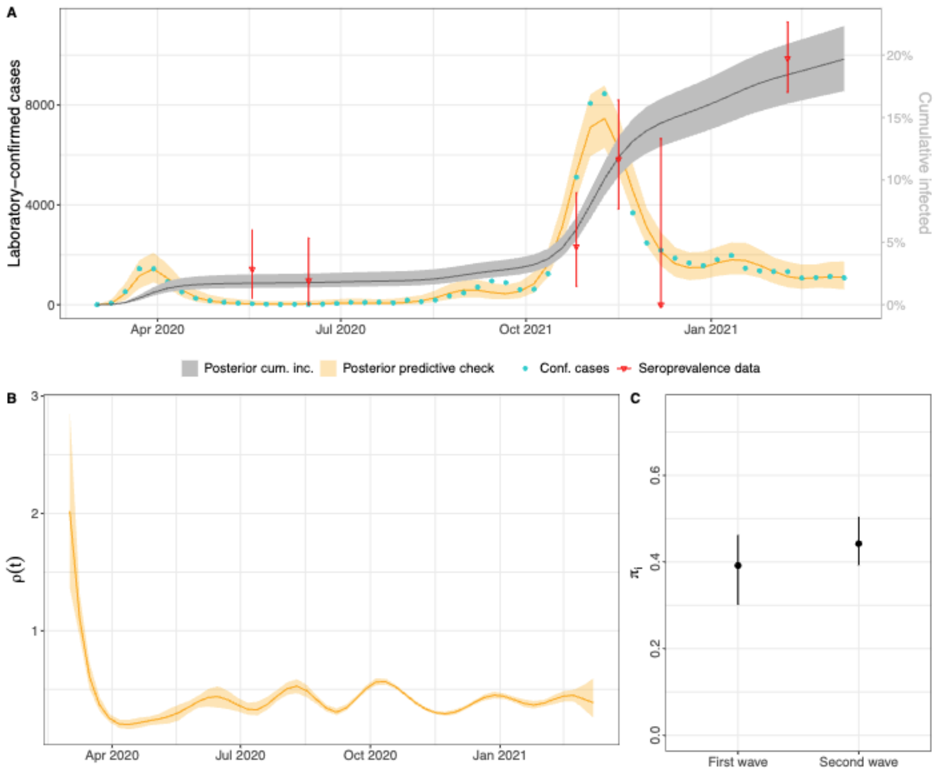

Modelled SARS-CoV-2 epidemic in Vaud, Switzerland. (A) Posterior predictive plot for laboratory-confirmed cases (left y-axis, orange ribbon) and cumulative incidence (right y-axis, gray ribbon). Green circles are weekly counts of laboratory-confirmed cases and red triangles show monthly seroprevalence estimates from data. (B) Estimates of the time-varying change in transmission rate using B-splines. (C) Estimated ascertainment rates for first and second wave.

A description of the data and methodology was added to the method section (lines 253-261).

Data from the canton of Vaud

For an additional example of our approach, we apply the B-spline based model to data from the canton of Vaud, Switzerland. The laboratory-confirmed SARS-CoV-2 cases are obtained from the Federal Office of Public Health. The serosurvey was performed by the Corona Immunitas Research Group in Switzerland [52]. As the seroprevalence data were collected over a prolonged period, we decided to use monthly seroprevalence estimates for fitting the model. We assumed two distinct ascertainment rates, one for the start of the epidemic until the 27th of July 2020 and one for after the 27th of July.

The obtained result figure is referenced in the result section (lines 370-373).

An additional example of the B-spline based model for non-stratified data can be found in S6 Fig. Here, the model is applied to real-world SARS-CoV-2 data from the canton of Vaud, Switzerland.

Reviewer #3: The authors have provided a careful example of a Bayesian workflow for a complex infectious disease modeling task. That is to say, they have presented a fitted model for a particular data set along with an iterative search of models, some of which may be applicable to related models for other data sets. I think this is a great way to go about

presenting modeling results.

The authors seem to have adequately addressed the concerns raised by the initial reviewers. My own suggestion would be for them to further explain what they mean by a Bayesian workflow, as the terms seems relatively uncommon at present. What are the benefits to this approach in general and can they explain some implications of their findings in creating the workflow, such as the relative efficiency of the different ODE solvers and time-varying functions?

We appreciate the reviewer's suggestion to improve our description of a Bayesian workflow. We added a definition of the Bayesian workflow to the introduction (lines 18-26).

Still, even the most basic situations require models of relatively high complexity, with many options available for each model feature, and difficulties of implementation and computational inefficiency limit the widespread adoption of these tools. In such situations, it is beneficial to describe the entire iterative process of model development, evaluation, and refinement, rather than presenting a single Bayesian model, since the optimal model choices frequently depend on the specifics of the real-world scenario. Gelman et al. (2020) have defined this comprehensive process as the Bayesian workflow, which utilizes simulated data to verify the accuracy of the inference and compare model efficiency [12].

As the reviewer suggests, our work has implications about the efficiency of the different solvers and time-varying functions in the context of compartmental transmission models. We have clarified that these considerations are the direct result of the Bayesian workflow in the discussion by reformulating the first sentence of the paragraph that presents this discussion (lines 394-396).

The Bayesian workflow, including the model comparisons we present, offers critical methodological insights into fitting compartmental transmission models to surveillance data.

A minor correction the authors should make is to provide explanations for the weighed error column in S1 Fig and S2 Fig.

We are thankful that the reviewer noticed this inconsistency and we have adjusted the descriptions of S1 Fig and S2 Fig.
